# Bilateral corticosteroid-induced osteonecrosis of the femoral head detected at a 6-week interval

**DOI:** 10.1186/s40064-015-1458-9

**Published:** 2015-11-02

**Authors:** Kazuhiko Sonoda, Takuaki Yamamoto, Goro Motomura, Satoshi Hamai, Kazuyuki Karasuyama, Yusuke Kubo, Yukihide Iwamoto

**Affiliations:** Department of Orthopaedic Surgery, Graduate School of Medical Sciences, Kyushu University, 3-1-1 Maidashi, Higashi-ku, Fukuoka, 812-8582 Japan

**Keywords:** Osteonecrosis of the femoral head, Magnetic resonance imaging, Corticosteroid, Bilateral

## Abstract

**Introduction:**

Corticosteroid-induced osteonecrosis of the femoral head (ONFH) often affects both femoral heads. Such bilateral ONFH cases are generally detected concurrently on magnetic resonance imaging (MRI). On the other hand, in unilateral cases, it is rare that contralateral ONFH is subsequently detected. We herein report a case in which bilateral ONFH was detected in both femoral heads by repeated MRI examination at an interval of 6 weeks.

**Case description:**

A 34-year-old man with purpura nephritis was started on corticosteroid therapy with prednisolone at 30 mg/day. Eight months after the initiation of corticosteroid therapy, he complained of left hip pain with no antecedent triggering activity. MRI obtained 8.5 months after the initiation of corticosteroid therapy showed the findings of osteonecrosis of the left femoral head, while no abnormalities were detected in the right femoral head. On the second MRI obtained 10 months after the initiation of corticosteroid treatment, however, osteonecrosis of the right femoral head was newly detected without an increase of the corticosteroid dose.

**Conclusions:**

This case may indicate that corticosteroid-induced bilateral ONFH do not always develop at the same time.

## Background

Nontraumatic osteonecrosis of the femoral head (ONFH) is considered to be the result of an ischemic event, leading to the death of osteocytes. Although several possible factors—hyperlipidemia, coagulation abnormalities, oxidative stress—have been suggested as the pathogenesis of ONFH, the precise mechanism remains unclear (Motomura et al. 2008; Nishida et al. 2008; Shuai et al. 2010; Ichiseki et al. 2011). Corticosteroid use is considered to be one of the major causative factors, and especially, high-dose corticosteroid therapy has been suggested as an important factor in the development of ONFH (Mont et al. 1997; Oinuma et al. 2001; Fukushima et al. 2010; Nakamura et al. 2010).

Corticosteroid-induced ONFH has been reported to develop early (1–2 months) after starting high-dose corticosteroid therapy, and the probability of affecting both femoral heads is considered to be approximately 80 % (Shigemura et al. 2012). In these bilateral cases, ONFH can be detected on magnetic resonance imaging (MRI) concurrently in the majority of cases (Oinuma et al. 2001; Fukushima et al. 2010). On the other hand, in patients with unilateral ONFH, it is rare that ONFH subsequently occurs on the contralateral side unless the corticosteroid dose had been increased. There is only one case report in which contralateral ONFH was detected at an intervals of 8 days, in which both ONFH were detected within 5 weeks after the initiation of corticosteroid therapy (Zhao et al. 2015).

We present a rare case in which ONFH was detected in both femoral heads at an interval of 6 weeks on MRI which were performed 8.5 and 10 months after the initiation of corticosteroid therapy. Written informed consent for publication of the case was obtained from the patient.

## Case description

A 34-year-old man (height 172 cm, weight 87 kg, body mass index 29.4 kg/m^2^) had been followed for purpura nephritis since he was 18 years of age. Since 32 years of age, he had been treated for hyperlipidemia with 20 mg of atorvastatin (cholesterol-lower agent). He had no history of alcohol abuse or smoking. Because of the gradual progression of renal failure, he was started on corticosteroid therapy (prednisolone at 30 mg/day) for the first time at 34 years of age.

**Fig. 1:**
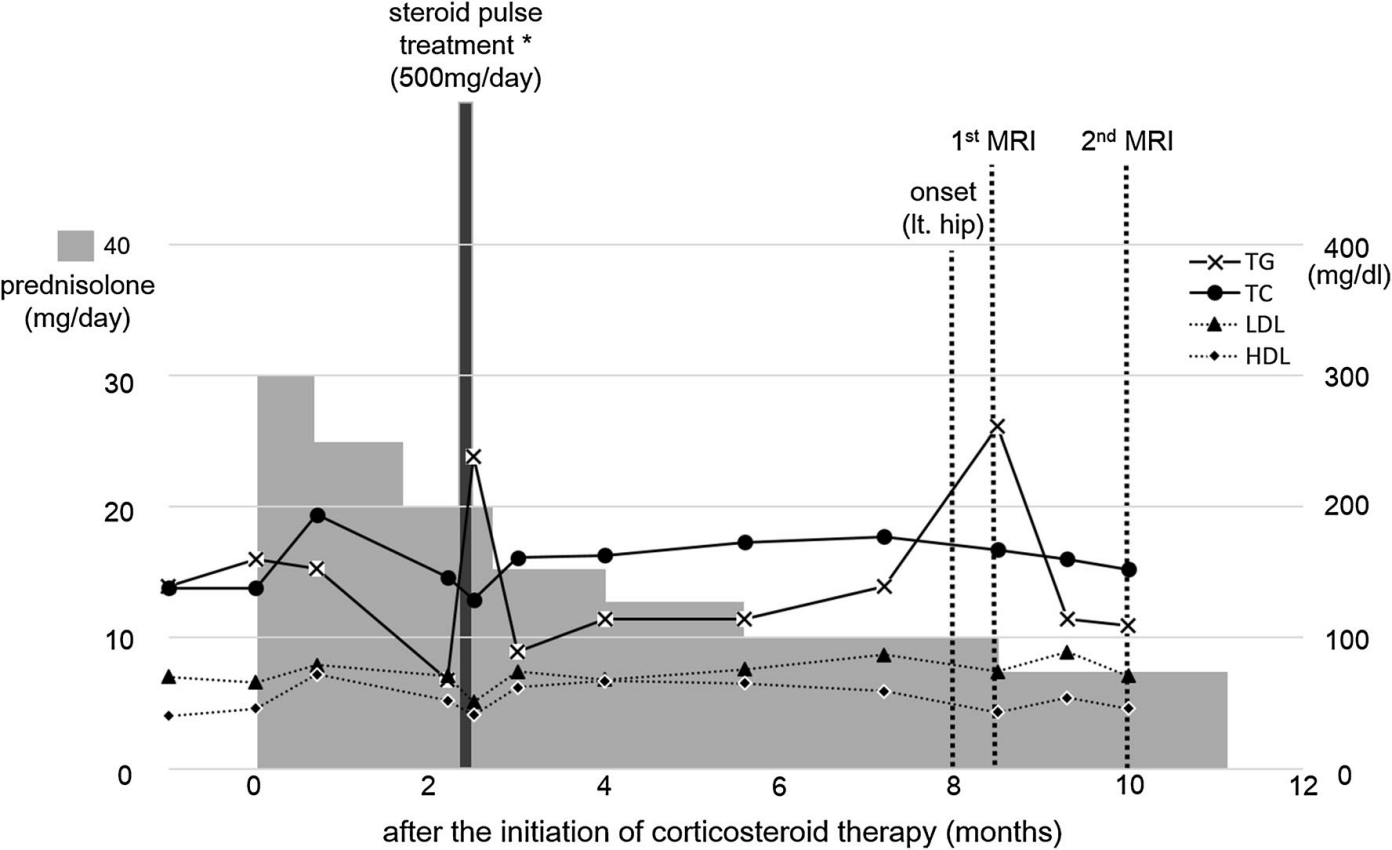
Time course for the prednisolone doses and the serum lipid levels. A 34-year-old man with purpura nephritis was started on corticosteroid therapy with prednisolone. Time course for the prednisolone doses and the serum lipid levels: triglycerides (TG), total cholesterol (TC), low-density lipoprotein cholesterol (LDL), and high-density lipoprotein cholesterol (HDL). * Corticosteroid pulsed treatment: methylprednisolone 500 mg/day × 6 days

The time course of the corticosteroid therapy is shown in Fig. 1. Two months after the initiation of corticosteroid therapy, he underwent tonsillectomy followed by corticosteroid pulsed treatment (500 mg of methylprednisolone for 6 days) for purpura nephritis. The prednisolone dose was gradually decreased, declining to 10 mg/day at 6 months after its initiation. He was not on any diet or exercise program during the corticosteroid therapy, even though he was overweight.

**Fig. 2:**
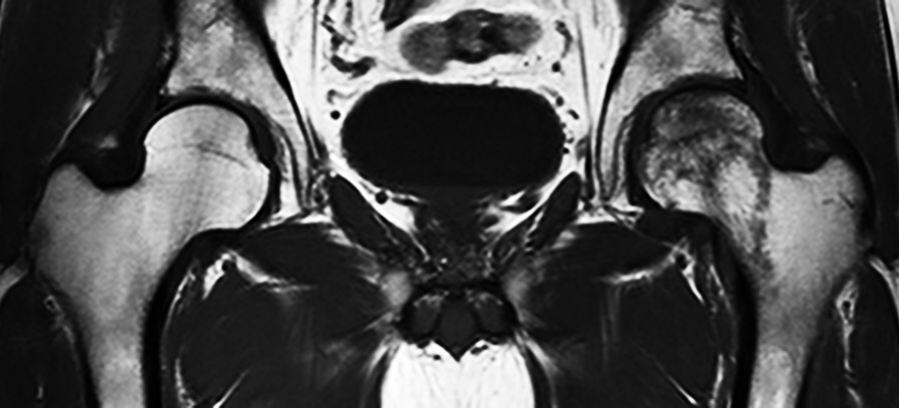
A coronal T1-weighted magnetic resonance image obtained 8.5 months after the initiation of corticosteroid therapy for purpura nephritis. Note the low-intensity band lesion in the left femoral head but no obvious abnormality in the right femoral head

**Fig. 3:**
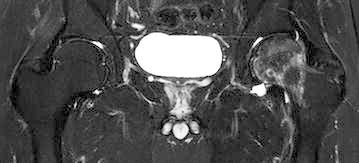
A coronal short-tau inversion recovery (STIR) image obtained 8.5 months after the initiation of corticosteroid therapy. Note the bone marrow edematous lesion distal to the band lesion on the left side, indicating femoral head collapse

Eight months after the initiation of corticosteroid therapy, he complained of left hip pain with no antecedent triggering activity. T1-weighted MRI scans obtained 2 weeks after the onset demonstrated a low-intensity band lesion in the left femoral head (Fig. 2). On the other hand, there was no obvious abnormality in the right femoral head. On short tau inversion recovery (STIR) images, an associated bone marrow edematous lesion was confirmed distal to the band lesion on the left side, indicating the occurrence of femoral head collapse (Fig. 3). The patient was diagnosed with unilateral osteonecrosis of the left femoral head and was referred to our department.

**Fig. 4:**
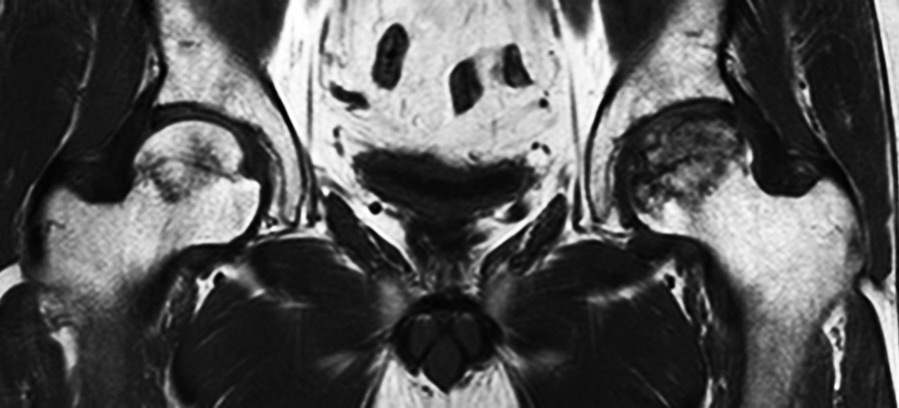
A coronal T1-weighted image obtained 10 months after the initiation of corticosteroid therapy (6 weeks after the first examination). Note the new T1 low-intensity band in the right femoral head

**Fig. 5:**
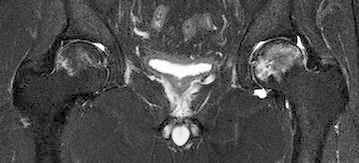
A coronal STIR image obtained 10 months after the initiation of corticosteroid therapy (6 weeks after the first examination). Edematous changes were not seen in the right femoral head

Although osteonecrosis of the left femoral head had collapsed already, joint preserving procedures, such as osteotomy and bone grafting, were considered to be more suitable than total hip arthroplasty since the patient was relatively young. For the purpose of evaluating the indications of osteotomy, he underwent MRI again 6 weeks after the initial MRI. This time, a low-intensity band newly appeared in the right femoral head on T1-weighted images (Fig. 4). STIR images did not reveal a bone marrow edema (Fig. 5). The second MRI scans demonstrated the new development of osteonecrosis in the right femoral head. As the right hip was asymptomatic and the patient did not will additional treatments, we decided to observe conservatively without restricting weight-bearing unless the right hip became symptomatic. Thereafter, his left hip underwent transtrochanteric anterior rotational osteotomy. Postoperatively, the patients started to perform active range of motion exercise 5 days after the operation and began to use wheelchairs at the same time. His left hip was maintained non-weight-bearing status until 5 week after the operation, followed by partial weight-bearing walking using two crutches. Full weight-bearing walking was permitted 4 months after the operation. His right hip had remained asymptomatic as of 8 months after the second MRI scans (7.8 months after the operation). At that time, the low intensity band was observed more obviously on T1-weighted images.

The time courses of the patient’s serum lipid levels (triglycerides, total cholesterol, low-density lipoprotein cholesterol, high-density lipoprotein cholesterol) are shown in Fig. 1. The serum level of triglycerides showed transient elevation twice during the period of observation (2.5 and 8.5 months after the initiation of corticosteroid therapy). The first elevation occurred immediately after corticosteroid pulse treatment, while the second occurred without obvious trigger events including the increase in the dose of corticosteroids. No other parameters showed significant elevations during the period of observation. There were also no remarkable changes in his coagulation parameters, hepatic enzymes, or C-reactive protein level.

## Discussion and evaluation

Oinuma et al. reported that corticosteroid-induced ONFH develops very early (1–2 months) after starting high-dose corticosteroid therapy, which could be detected at least 3 months after the initiation of treatment in all the cases (Oinuma et al. 2001). In the current case, it is considered that osteonecrosis of the left femoral head had developed early after either high-dose oral corticosteroid therapy (30 mg/day) or the corticosteroid pulsed treatment. In the right side, however, no evidence of osteonecrosis could be detected on the first MRI obtained 6 months after corticosteroid pulse treatment, indicating the difference in timing of the developing osteonecrosis.

It is rare that contralateral ONFH newly develops in unilateral cases. To our knowledge, there are two case reports presenting new corticosteroid-induced ONFH developing in the contralateral femoral head. In one case, the corticosteroid dose had been increased to more than 30 mg/day before ONFH developed in the contralateral hip (Sonoda et al. 2015). In another case, new contralateral ONFH was detected on MRI at an interval of 8 days within 5 weeks after the initiation of corticosteroid therapy, indicating that new contralateral ONFH developed immediately after the initiation of corticosteroid treatment (Zhao et al. 2015). In contrast, in the current case, the first MRI presenting no evidence of osteonecrosis in the contralateral hip was performed at 8.5 months after the initiation of corticosteroid therapy (6 months after the corticosteroid pulse treatment).

It remains unclear why new ONFH developed in the current case, however, one possible explanation may be the hyperlipidemia. The serum level of cholesterol showed no remarkable changes while that of triglycerides had elevated twice after the initiation of corticosteroid therapy; the first elevation occurred immediately after corticosteroid pulse treatment, and the second occurred 8.5 months after the initiation of corticosteroid therapy. A previous study suggested that hypertriglyceridemia was associated with the development of ONFH (Bhojwani et al. 2014). In animal models of corticosteroid-induced osteonecrosis, corticosteroid-induced hypertriglyceridemia was suggested to be associated with the development of osteonecrosis (Yamamoto et al. 1997; Kabata et al. 2005; Motomura et al. 2008; Pengde et al. 2008). In the current case, the second elevation of serum level of triglycerides may thus be associated with the development of contralateral ONFH. The patient had received a cholesterol-lower agent, however, a triglyceride-lower agent had never been prescribed.

## Conclusions

Bilateral ONFH generally develops simultaneously after the initiation of corticosteroid therapy. Considering the current case, however, it should be noted that corticosteroid-induced bilateral ONFH do not always developed at the same time.
